# Suspected allergy to Beta-Lactam antibiotics: An infectiological perspective 

**DOI:** 10.5414/ALX02314E

**Published:** 2022-02-01

**Authors:** Cord Sunderkötter

**Affiliations:** Department of Dermatology and Venereology, University Hospital Halle, Martin-Luther-University Halle-Wittenberg, Halle, Germany

**Keywords:** bacterial resistance to antibiotics, penicillin, cefazolin, beta-lactam allergy, skin and soft-tissue infections, erysipelas, cellulitis, Clostridium difficile, desensitization

## Abstract

The administration of alternative broad-spectrum antibiotics because of a suspected allergy to beta-lactam antibiotics (BLA) is one reason for the increase in bacterial resistance to antibiotics and results in further problems, such as reduced efficiency against the causative bacteria, longer hospital stays, higher prices, and more adverse events. Patients with documented BLA allergy experience *Clostridium difficile* infections and postoperative surgical-site infections more frequently than patients without this label. Yet, in cases of documented and even proven IgE-mediated allergy to a BLA, such as penicillin or cephalosporin, the careful application of a different BLA with dissimilar core and side chains is possible. Cefazolin, e.g., would often be a candidate for skin and soft-tissue infections (e.g., cellulitis) or for perioperative prophylaxis, because it does not share a common side chain with any other BLA and tackles most causative bacteria. In case of severe cellulitis, a carbapenem would be a candidate. After type IV-reactions (benign maculopapular rash), an infectiologist’s choice would be to apply another narrow-spectrum BLA. In cases where a long-lasting therapy with penicillin is indicated (e.g., for late syphilis or prophylaxis of erysipelas) in presence of a proven IgE-mediated allergy, desensitization would be the infectiologist’s choice.

## Introduction 

The proportion of bacteria resistant to many antibiotics is continuously increasing. Even though headline-producing estimates of maximum numbers are not always reliable – such as the assumption that 10 million people worldwide will die annually from infections with antibiotic-resistant pathogens by 2050 – they at least give us an idea of the health burden and problems we will face in the long term [1]. One cause for the increase in antibiotic resistance is the high number of suspected allergies to beta-lactam antibiotics (BLA) and the ensuing prescription of alternative antibiotics. This procedure results in yet other problems or dangers for patients and the healthcare system. 

## Impact on bacterial antibiotic resistance 

It is undeniable that prescribing broad-spectrum antibiotics instead of BLAs with narrower pathogen spectrums (penicillin or first- or second-generation (or group 1 and 2) cephalosporins) leads to higher rates of resistant bacteria in humans. 

This is a general phenomenon, regardless of whether the prescription of such alternative antibiotics occurs due to confirmed or suspected BLA allergy or for other reasons. With regard to resistance developing specifically as a consequence of BLA allergy, there have only been a few studies. One demonstrated a 14% higher prevalence of methicillin-resistant *S. aureus* (MRSA) and a 30% higher prevalence of glycopeptide- or vancomycin-resistant enterococci (VRE) in hospitalized patients with confirmed or presumed penicillin allergy, compared to those without [2]. Another study demonstrated that patients with suspected BLA allergy had a 69% increased incidence of MRSA, and that 55% of these cases were due to the administration of BLA-alternative antibiotics, then often with a very broad spectrum [3]. 

## Impact on affected patients 

### More adverse effects, lower efficacy, longer inpatient stays 

BLAs are the best antibiotics for both oral [4] and parenteral therapy [5] of skin and soft-tissue infections. 

However, patients with confirmed or even suspected hypersensitivity to a penicillin or a cephalosporin do not receive BLAs, which have a narrow spectrum and low side effects, but rather an antibiotic from another class or group (e.g., clindamycin, fluoroquinolones, or even vancomycin, gentamicin) with the consequence of not only a broader spectrum, but also of lower efficacy against the causative bacteria, longer inpatient stays, a higher price, and an increased rate of adverse events [6, 7, 8, 9]. 

Corresponding studies have revealed that even when a BLA was clearly indicated and the reported alleged allergic reaction was weak, with a low probability for an IgE-mediated type I reaction, and where an alternative BLA would have been possible, alternative antibiotics were still given, with all the aforementioned disadvantages for the affected patients [3, 10]. According to a Canadian study, 95 (19%) of 507 hospitalized patients had reported a BLA allergy; as a result, 25 (35%) of them did not receive an alternative BLA, not even when the alleged allergic symptoms had not been severe or serious (in 13 patients). As a result of the antibiotics that were eventually administered, the risk of an adverse event became three-fold higher [11]. 

### Healthcare associated infections 

Data from the United States show that the prevalence of antibiotic-associated overgrowth and infection with *Clostridium difficile* was 23% higher in hospitalized patients with a reported penicillin allergy than in patients without such a medical history [12]. Similarly, a study from the United Kingdom demonstrated that among patients with penicillin allergy, the incidence for infection with *Clostridium difficile* was 25% higher after adjusting for other confounding factors. More than 10% of cases in this cohort were due to the administration of fluoroquinolones in place of BLA [3]. 

### Perioperative antibiotic prophylaxis 

Surgical-site infections account for nearly half of healthcare-associated infections [13]. In cases where perioperative antibiotic prophylaxis is therefore indicated, BLAs are most commonly recommended internationally, in particular cefazolin, a group-1 cephalosporin active against *S. aureus*, and cefoxitin, for gastrointestinal or gynecologic procedures. However, patients with suspected BLA allergy were more likely than others to receive clindamycin or even vancomycin or gentamicin instead of cefazolin, as demonstrated by a study on 8,385 patients in the United States; the rate of surgical-site infections was 50% higher under such antibiotics (odds ratio: 1.51) [14]. In addition, more postoperative *Clostridium difficile* infections were observed as a consequence of perioperative antibiotic prophylaxis with such alternative antibiotics (clindamycin or vancomycin) [15]. Also, non-IgE-mediated reactions occurred more frequently after perioperative prophylaxis in patients with reported BLA allergy [16]. 

### Procedure in cases of suspected BLA allergy and acute need for antibiotic therapy 

If in the medical history a reaction is reported within 1 – 6, possibly 72 hours after administration of an antibiotic or if symptoms of a type I immune reaction, i.e., anaphylactic symptoms, such as pruritic wheals, angioedema, drop in blood pressure, bronchoconstriction, rhinitis, stridor, have occurred, then caution is advised. 

If the patient can remember the antibiotic, and it was a penicillin or a cephalosporin, but no other details are known, then penicillins and all BLA with similar side chain (mostly cephalosporins) must be avoided. It is worth asking whether a BLA has been given again afterwards and was tolerated without reactions; this is sometimes the case and would justify giving this BLA again in an acute situation. 

When antibiotic therapy cannot be delayed, as in the case of life-threatening infections, the fact that carbapenems and monobactams are good potential alternatives for patients with confirmed penicillin and cephalosporin allergy helps. Contrary to previous reports, a systematic review determined that even though a small number (36 of 838) of patients with a proven or possibly IgE-mediated reaction to penicillin also reported or showed a suspected reaction to a carbapenem, the clinical signs would have been consistent with an IgE-mediated reaction to carbapenem in only 20 patients, and that ultimately in only one of these cases a type I allergy was actually confirmed or proven. The authors therefore recommended that carbapenems can be given in patients with BLA allergy, albeit with precautions, by giving the first dose cautiously (fractionated or slowly, e.g., with 1/10^th^ of the dose as a starting dose, then 2 hours later with a full dose [17]) and under medical supervision according to the conditions of a provocation test [18]. 

However, it is even possible to use a BLA with a side chain different from the one of the accused preparation. Cefazolin, which has no related side chains to other BLAs, is often suitable for this purpose. A prospective study found no cross-reactivity with cefazolin, cefuroxime, and ceftriaxone in patients with proven penicillin allergy based on skin testing or provocation [19]. Accordingly, the American Academy of Pediatrics has stated that the likelihood of a patient with penicillin allergy also reacting to a cephalosporin with a different side chain is no higher than in patients in whom no penicillin allergy was previously known [20]. A corresponding meta-analysis found a very low cross-reaction with cefazolin in patients with presumed penicillin allergy and did not exclude the use of cefazolin even in cases of explicitly proven IgE-mediated penicillin allergy. However, administration should then only proceed under appropriate precautions under which an anaphylactic reaction can be controlled [21]. The German AWMF guideline on the diagnosis of suspected BLA hypersensitivity expresses it somewhat more cautiously, but also recommends the administration of a BLA with low risk of cross-reactivity under the precautions of a provocation test in cases of an urgent indication and after individual case assessment. It also provides online dosing suggestions on the AWMF website [22]. 

Due to the low risk of cross-reactions with preparations with different side chains even within one cephalosporin generation or group, it is now explicitly discouraged to pursue the previously frequently used strategy of switching to the more broadly effective group 3 or 4 cephalosporins in the case of alleged BLA allergy in cases where group 1 or 2 cephalosporins would be indicated, since the associated higher rates of *Clostridium difficile* infection are more threatening for the patient than the risk of an allergic reaction [2], especially since the antibacterial spectrum of the different cephalosporin groups is very different. 

For cefuroxime, although there are also few inferred cross-reactions with penicillin and other BLAs, the reported cross-reactions with cefoxitin and also other methoxyimino cephalosporins (e.g., ceftriaxone, cefotaxime) [23, 24] may have led to it not being listed as an alternate antibiotic for BLA in relevant reviews [9, 17, 28] (apart from that, it should be given parenterally rather than orally because of unreliable absorption). 

If the medical history clearly shows that the previous reaction to a BLA (or other antibiotic) was an uncomplicated, morbilliform or maculopapular exanthema that occurred only 72 hours after the initial administration of the BLA, i.e., it was most likely a T-cell-mediated drug reaction, one can use another BLA first in many clinical situations; just as, when such mild reactions occur for the first time, the suspected BLA may be continued (“through-treated”) if necessary. 

In the case of intolerance reactions to a BLA, such as diarrhea, nausea, vomiting, or headache, depending on the extent of the reaction and the patient’s medical history, an anew administration of the suspected BLA or at least another BLA would not be excluded. 

Whenever, in the case of a proven IgE-mediated allergy to a BLA, a prolonged treatment with penicillin or another narrow-spectrum BLA is planned and represents the drug of choice from an infectiological point of view (e.g., for daily treatment of neurosyphilis or for prophylaxis of erysipelas with penicillin each day), acute desensitization and then further continuous administration is possible and recommended (in the case of T-cell-mediated reaction, however, desensitization has no reliable effect according to the current knowledge). Acute desensitization is not appropriate from an infectious point of view in cases of suspected sepsis, severe complicated soft tissue infections (e.g., with neutropenia), or other severe infections (e.g., meningitis), because in these cases even a slight delay of an adequately dosed antibiotic administration is associated with a worsening of the prognosis. 

There are well-tabulated algorithms for this procedure in the German and international literature [9, 17, 27]. The article by Querbach et al. [17] contains good checklists for diagnostic and therapeutic measures; however, I would restrict the general recommendation to switch to a cephalosporin of group 3 – 5 in the case of penicillin allergy, since in many indications, such as limited cellulitis or erysipelas, a too broad and incorrect bacterial spectrum would be addressed, with the adverse consequences already mentioned (e.g., causing a *Clostridium difficile* infection [2]). 

## Examples 

### Patient with a medical history of reaction to penicillin or ampicillin, most likely type I reaction, but maculopapular exanthema not excluded 

In a patient with a medical history of suspected type I reaction to, e.g., penicillin or ampicillin in the last 5- 10 years with limited cellulitis (even after 10 years, but after such a long, time type I reactions are often difficult to detect without intermediate re-exposure), parenteral therapy with cefazolin would be possible [9, 21, 28] as it is effective against *S. aureus* and recommended for limited cellulitis in guidelines [5, 25] . Cefazolin would be better from an infectious-disease perspective than the macrolides or clindamycin (both of which would also be indicated for the case of penicillin allergy) [5]. To be on the safe side, the first administration should be done cautiously (e.g., fractionated or slowly, e.g., with 1/10 of the dose as a starting dose, then 1/3 of the dose, and then the target dose at intervals of at least 30 minutes, or so that the full dose is reached after 1.5 – 2 hours [17]) and under conditions in which an anaphylactic reaction would be manageable (such as in provocation testing) [9]. If there is a transition to severe cellulitis, carbapenem under the above conditions would also be possible, if appropriate. An allergological skin test would be desirable but would only be possible in very few cases in this situation. By contrast, flucloxacillin (otherwise a first-line agent for limited cellulitis), ampicillin, piperacillin, ticarcillin, cefadroxil (otherwise a first-line oral agent), cephalexin (otherwise a first-line oral agent), cefaclor, and cefprozil would be contraindicated in this case [27]. Cefazolin, meanwhile, would also be an option if it is not clear whether the patient’s reaction was not perhaps an uncomplicated morbilliform or maculopapular exanthema after oral ingestion of a penicillin or a cephalosporin. While it is recommended to avoid the same drug in this case, in my experience, many patients at least remember whether they had taken the drug orally or not – in the former case it could not have been cefazolin. On the other hand, cefazolin and other BLAs would also be contraindicated if such a patient has a history of symptoms of DRESS, Stevens Johnson syndrome, or even TEN after taking penicillin, ampicillin, or another cephalosporin, or has subsequently suffered from interstitial nephritis, serum sickness, immune hepatitis, or hemolytic anemia. In such diseases, any provocation testing is also prohibited. In these patients, in the case of limited cellulitis, clindamycin, or a macrolide (preferably after antibiogram) should be used. 

As an aside, serum sickness is the classic systemic reaction to large immune complexes, such as occur when approximately equimolar concentrations of antigen (antibiotic) and antibody meet in the blood [25]. In contrast, additional factors are involved in the more common IgA vasculitis in context with intake of antibiotics, such as abnormally hypogalactosidated IgA [26]. Nevertheless, even in the case of IgA vasculitis after administration of a BLA, I would first refrain from re-administration of the same BLA or a BLA with a similar side chain – yet not from all BLAs. 

### Patients with limited cellulitis (or erysipelas) and medical history of suspected type I reaction to a cephalosporin 

In a patient with limited cellulitis (or erysipelas) and anamnestic suspicion of a type I reaction to ampicillin, amoxicillin, cefaclor, and cefprozil, one must avoid cephalosporins with similar side chains in acute cases without desensitization (i.e., ampicillin, amoxicillin, cefaclor, and cefprozil), but may instead choose a penicillin or cephalosporin with a different side chain, such as cefazolin, and administer it as a stepwise provocation test under monitoring. From an infectiological point of view, the latter is usually more effective and better than the non-BLA macrolides or clindamycin, which are also possible agents in this case. It is also helpful to note here that it may not have been any of the often suspected orally given antibiotics. In severe cellulitis, a carbapenem would also be possible. 

## Conclusion 

In cases of presumed allergy to BLAs and indication for antibiotic treatment, one should not abandon all BLAs because administration of an alternative broad-spectrum antibiotic leads to more bacterial antibiotic resistance, lower efficacy against causative bacteria, longer hospital stays, higher prices, and more adverse effects.

- Prudent administration of a BLA with a side chain differing from that of the suspected antibiotic is possible.
- For skin and soft tissue infections (erysipelas, phlegmon) or for perioperative prophylaxis, cefazolin is often an option, because it has no related side chain with other BLAs and due to its intravenous route is not one of the often suspected or accused oral antibiotics. For severe cellulitis, a carbapenem would be possible.
- After uncomplicated type IV reactions (maculopapular exanthema), another BLA may also be used.
- In the case of planned, prolonged treatment with, e.g., penicillin (e.g., neurosyphilis, erysipelas prophylaxis) and proven type I allergy, desensitization is recommended from an infectiological point of view.

## Acknowledgment 

I would like to thank Dr. Gerda Wurpts, Dr. Burkhard Kreft, and Prof. Dr. Knut Brockow for their valuable suggestions to improve this manuscript. 

## Funding 

No funding for this manuscript. 

## Conflict of interest 

No direct conflict of interest concerning the contents of this manuscript, but participation at advisory boards or consulting activity for the companies Bayer AG, Biotest AG, Infectopharm, Janssen, Pfizer. 
